# Association between *XPG* polymorphisms and stomach cancer susceptibility in a Chinese population

**DOI:** 10.1111/jcmm.12773

**Published:** 2016-01-28

**Authors:** Yun‐Zhi Chen, Fang Guo, Hong‐Wei Sun, Hong‐Ru Kong, Sheng‐Jie Dai, Shi‐Hao Huang, Wen‐Wei Zhu, Wen‐Jun Yang, Meng‐Tao Zhou

**Affiliations:** ^1^ Department of General SurgeryFirst Affiliated HospitalWenzhou Medical UniversityWenzhouZhejiangChina; ^2^ Department of Gynecology and ObstetricsPeople's HospitalWenzhouZhejiangChina; ^3^ Department of General SurgeryHuashan HospitalFudan University Shanghai Cancer CenterShanghaiChina

**Keywords:** stomach cancer, *XPG*, polymorphism, genetic susceptibility

## Abstract

Xeroderma pigmentosum group G (*XPG*) protein plays an important role in the DNA repair process by cutting the damaged DNA at the 3′ terminus. Previous studies have indicated some polymorphisms in the *XPG* gene are associated with stomach cancer susceptibility. We performed this hospital‐based case–control study to evaluate the association of four potentially functional *XPG* polymorphisms (rs2094258 C>T, rs751402 C>T, rs2296147 T>C and rs873601G>A) with stomach cancer susceptibility. The four single nucleotide polymorphisms (SNPs) were genotyped in 692 stomach cancer cases and 771 healthy controls. Logistic regression analysis was conducted, and odds ratios (ORs) and 95% confidence intervals (CIs) were used to assess the association of interest. Of the studied SNPs, *XPG* rs873601G>A polymorphism was found to significantly associate with stomach cancer susceptibility (AA *versus* GG/AG: OR = 1.31, 95% CI = 1.03–1.66, *P* = 0.027). Combined analysis of all SNPs revealed that the individuals with two of risk genotypes had a significantly increased stomach cancer risk (OR = 1.52, 95% CI = 1.13–2.06). In the stratification analysis, the association between the rs873601AA genotype and stomach cancer risk was observed in older group (>59 year), as well as patients with non‐cardia stomach cancer. Further combined analysis indicated men, smokers, or non‐drinkers more than one risk genotypes had a significantly increased stomach cancer risk. Our results indicate that *XPG* rs873601G>A polymorphism may be associated with the risk of stomach cancer. Further prospective studies with different ethnicities and large sample sizes are needed to validate our findings.

## Introduction

Cancer poses an substantial burden on society worldwide, and the incidence rate of cancer remains rising due to the growth of ageing populations and increasing exposure to well‐established risk factors, such as smoking, overweight, and physical inactivity. According to GLOBOCAN estimates, there were approximately 14.1 million new cases and 8.2 million new cancer‐related deaths that occurred in 2012 worldwide 1. Among them, about 951,600 new stomach cancer cases and 723,100 deaths occurred in 2012. Stomach cancer is the third most common cause of cancer‐related death, just next to lung cancer and liver cancer that led to 1,589,900 and 745,500 death respectively. The highest risk area involves the Eastern Asia (including China), Central and Eastern Europe, and South America, while the Northern America and most parts of Africa are the lowest risk area 1. The risk factors of stomach cancer include the consumption of salted and nitrated foods, cigarette smoking, low intake of fresh fruits and vegetables 2. Overweight and obesity may be a risk factor for residents in Western countries 3, but not for Chinese subjects 4. *Helicobacter pylori* infection is an important and well‐established aetiologic factor for stomach cancer in all populations worldwide, and the infected individuals by *H. pylori* show about 3‐ to 6‐fold increased risk of developing stomach cancer 5. However, even in the regions with a high prevalence of *H. pylori* (*e.g*., South America, Asia, Africa and Eastern Europe), only a small proportion of individuals eventually develop stomach cancer. Taken together, it suggests that environmental factors are necessary, but not sufficient to cause cancer, and genetic susceptibility may play a critical role in the tumorigenesis of stomach cancer 6.

DNA repair genes play important roles in maintaining the stability and integrity of genome. More than 130 genes are involved in the different DNA repair pathways, including nucleotide excision repair (NER) pathway 7. The NER pathway is the primary mechanism for the removal of DNA adducts and lesions caused by chemical adducts, and thus is an important part of the cellular defense against a large variety of structurally unrelated DNA lesions 8, 9. In humans, xeroderma pigmentosum group G (XPG) is one of the seven XP complementation groups (XPA to XPG) that have been identified 10. It functions as an endonuclease that can cut the damaged DNA at the 3′ sites of the lesion during the process of DNA repair 11.

As so far, most of the studies that investigated the association between polymorphisms in the *XPG* gene and cancer susceptibility have focused on rs17655 C>G polymorphism and results are not conclusive 12. Moreover, studies on stomach cancer were very few. There were only two studies having examined the association of *XPG* rs17655 C>G with stomach cancer risk 13, 14. In addition, three studies investigating potentially functional single nucleotide polymorphisms (SNPs) and stomach cancer risk were published more recently 15, 16, 17. Therefore, it is imperative to identify new susceptibility loci in the *XPG* gene for stomach cancer. In this study, we selected four potentially functional SNPs (rs2094258 C>T, rs751402 C>T, rs2296147 T>C and rs873601G>A) and explored their association with stomach cancer susceptibility in a hospital‐based case–control study with 692 stomach cancer cases and 774 healthy controls.

## Materials and methods

### Study population

The current case–control study consisted of 692 stomach cancer cases and 774 healthy controls recruited from the First Affiliated Hospital of Wenzhou Medical University between January 2010 and September 2013. Details of the characteristics of the subjects in this case–control study were described previously 18. All cases were histologically/pathologically confirmed by two experienced pathologists. All the 774 healthy controls were individuals receiving the health screening in the same hospital, and they were frequency‐matched to the cases by gender and age distribution. All of the participants provided written informed consent. This study was approved by the Clinical Research Ethics Committee of Wenzhou Medical University.

### DNA extraction and genotyping

Genomic DNA used for the assay was extracted from peripheral blood samples as described previously 16. We chose the three potentially functional SNPs (rs2094258 C>T, rs2296147 T>C and rs873601G>A) following a previously published protocol 16. We also chose the rs751402 C>T that is located in the 5′ UTR region which was reported in another study 15. Taqman real time PCR method was performed to detect the genotypes of the four selected potentially functional SNPs by using a 7900 HT sequence detector system (Applied Biosystems, Foster City, CA, USA). PCR reactions were carried out in 384 wells with a total volume of 5 μl containing 10 ng of genomic DNA for each SNP. At least 10% of the samples were randomly selected and re‐genotyped to ensure the accuracy of the analysis.

### Statistical analysis

Chi‐squared test were performed to examine the differences in the distribution of various characteristics and genotype frequencies between the stomach cancer cases and the healthy controls. Goodness‐of‐fit chi‐squared test was performed to assess the Hardy–Weinberg equilibrium (HWE) for each SNP by comparing observed and expected genotype frequencies. Odds ratios (ORs) and their corresponding 95% confidence intervals (CIs) were calculated to evaluate the strength of the association between these four SNPs and stomach cancer risk by logistic regression. We managed all the statistical analysis by using SAS software (version 9.1; SAS Institute, Cary, NC, USA). A two‐sided *P*‐value less than 0.05 was considered to be statistically significant.

## Results

### Population characteristics

All the stomach cancer cases and healthy controls were from Chinese Han descent. The characteristics of the study population are shown in Table S1. Three samples in controls failed in spite of repeated genotyping tests, thus a total of 692 stomach cancer patients and 771 healthy controls were included in the final analysis. The mean age (±SD) for case and control groups was 59.22 (±11.05) and 59.71 (±11.35) years respectively. Cases consisted of 492 men and 200 women, while healthy controls included 546 men and 225 women. No significant difference was observed in the age and gender distribution between two groups (*P* = 0.864 and *P* = 0.906 respectively). Compared to the healthy controls, the stomach cases were more likely to be non‐smokers (*P* < 0.0001), non‐drinkers (*P* = 0.0005) as well as nutrient deficient and with lower BMI (*P* < 0.0001).

### Distributions of selected SNPs and risk of stomach cancer

The genotypes and allele frequencies of the four polymorphisms were shown in Table 1. The observed genotype frequency distributions of the four polymorphisms were in consistent with HWE for controls (*P* = 0.803 for rs2094258 C>T, *P* = 0.416 for rs751402 C>T, *P* = 0.535 for rs2296147 T>C and *P* = 0.415 for rs873601G>A). The significant differences in genotype frequency distribution were only observed for the rs873601G>A polymorphism between cases and controls. Compared with carriers of the rs873601GG/AG genotypes, carriers of the AA genotype had a significantly increased stomach cancer risk (OR = 1.31, 95% CI = 1.03–1.66, *P* = 0.027). We also found that the presence of two risk genotypes significantly conferred increased stomach cancer risk (OR = 1.52, 95% CI = 1.13–2.06, *P* = 0.006, and adjusted OR = 1.44, 95% CI = 1.03–2.01, *P* = 0.035). Moreover, the risk of developing stomach cancer was also significantly increased among those carrying one or more risk genotypes (OR = 1.32, 95% CI = 1.04–1.69, *P* = 0.025).

**Table 1:** Logistic regression analysis of associations between the genotypes of *XPG* and stomach cancer susceptibility in a Chinese population

Genotype	Cases (*N* = 692)	Controls (*N* = 771)	*P* a	Crude OR (95% CI)	*P*	Adjusted OR (95% CI)^b^	*P* b
rs2094258 C>T							
CC	287 (41.47)	291 (37.74)	0.296^c^	1.00		1.00	
CT	304 (43.93)	368 (47.73)		0.84 (0.67–1.05)	0.119	0.85 (0.68–1.06)	0.150
TT	101 (14.60)	112 (14.53)		0.91 (0.67–1.25)	0.577	0.92 (0.67–1.26)	0.601
Additive				0.93 (0.80–1.08)	0.313	0.93 (0.80–1.08)	0.347
Dominant	405 (58.53)	480 (62.26)	0.145^d^	0.86 (0.69–1.06)	0.145	0.86 (0.70–1.07)	0.177
Recessive	591 (85.40)	659 (85.47)	0.970^e^	1.01 (0.75–1.35)	0.970	1.00 (0.75–1.35)	0.980
rs751402 C>T							
CC	286 (41.33)	351 (45.53)	0.227^c^	1.00		1.00	
CT	313 (45.23)	331 (42.93)		1.16 (0.93–1.45)	0.184	1.17 (0.91–1.49)	0.221
TT	93 (13.44)	89 (11.54)		1.28 (0.92–1.78)	0.140	1.11 (0.77–1.61)	0.576
Additive				1.14 (0.98–1.33)	0.088	1.09 (0.92–1.29)	0.335
Dominant	406 (58.67)	420 (54.47)	0.106^d^	1.19 (0.96–1.46)	0.106	1.15 (0.92–1.45)	0.226
Recessive	599 (86.56)	682 (88.46)	0.273^e^	1.19 (0.87–1.62)	0.272	1.03 (0.73–1.46)	0.872
rs2296147 T>C							
TT	442 (63.87)	475 (61.61)	0.464^c^	1.00		1.00	
CT	217 (31.36)	264 (34.24)		0.88 (0.71–1.10)	0.273	0.93 (0.73–1.19)	0.562
CC	33 (4.77)	32 (4.15)		1.11 (0.67–1.83)	0.688	1.38 (0.78–2.44)	0.268
Additive				0.95 (0.80–1.14)	0.586	1.03 (0.84–1.25)	0.809
Dominant	250 (36.13)	296 (38.39)	0.371^d^	0.91 (0.73–1.12)	0.372	0.97 (0.77–1.23)	0.823
Recessive	659 (95.23)	739 (95.85)	0.567^e^	1.16 (0.70–1.90)	0.566	1.41 (0.81–2.48)	0.227
rs873601G>A							
GG	172 (24.86)	205 (26.59)	0.087^c^	1.00		1.00	
GA	333 (48.12)	396 (51.36)		1.00 (0.78–1.29)	0.986	1.08 (0.81–1.42)	0.604
AA	187 (27.02)	170 (22.05)		1.31 (0.98–1.75)	0.067	1.33 (0.96–1.84)	0.086
Additive				1.14 (0.99–1.32)	0.071	1.15 (0.98–1.36)	0.088
Dominant	520 (75.14)	566 (73.41)	0.449^d^	1.10 (0.87–1.39)	0.450	1.15 (0.89–1.50)	0.287
Recessive	505 (72.98)	601 (77.95)	0.027^e^	**1.31 (1.03–1.66)**	**0.027**	1.27 (0.97–1.65)	0.084
Combined effect of risk genotypes							
0	145 (20.95)	200 (25.94)	0.023^c^	1.00		1.00	
1	367 (53.03)	408 (52.92)		1.24 (0.96–1.60)	0.099	1.20 (0.90–1.60)	0.211
2	180 (26.01)	163 (21.14)		**1.52 (1.13–2.06)**	**0.006**	**1.44 (1.03–2.01)**	**0.035**
0	145 (20.95)	200 (25.94)		1.00		1.00	
1–2	547 (79.05)	571 (74.06)	0.025	**1.32 (1.04–1.69)**	**0.025**	1.27 (0.97–1.66)	0.088

a Chi‐squared test for genotype distributions between stomach cancer cases and controls.

b Adjusted for age, gender, BMI, smoking and drinking status.

c Additive models.

d Dominant models.

e Recessive models.

OR, odds ratio; CI, confidence interval.

The results were in bold, if the 95% CI excluded 1 or P<0.05.

### Stratification analysis

We further explored the association between *XPG* polymorphisms and stomach cancer risk in stratification analysis by age, gender, smoking status, pack‐year, drinking status, BMI and tumour sites (Table 2). First of all, we only found that the rs873601 AA genotype was shown to significantly increase stomach cancer risk among the older subjects (adjusted OR = 1.54, 95% CI = 1.05–2.25, *P* = 0.028) and the non‐cardia subjects (adjusted OR = 1.36, 95% CI = 1.01–1.82, *P* = 0.041). We also observed a borderline significantly increased stomach cancer risk for the men (adjusted OR = 1.37, 95% CI = 1.00–1.89, *P* = 0.052) and ever smokers (adjusted OR = 1.43, 95% CI = 0.95–2.15, *P* = 0.085). We next combined the four SNPs, and found that the presence of one or two risk genotypes was significantly associated with increased stomach cancer risk among men (adjusted OR = 1.40, 95% CI = 1.01–1.93, *P* = 0.043), ever smokers (adjusted OR = 1.83, 95% CI = 1.20–2.80, *P* = 0.005), subjects with pack‐years ≤27 (adjusted OR = 1.83, 95% CI = 1.02–3.23, *P* = 0.045) and never drinkers (adjusted OR = 1.41, 95% CI = 1.02–1.94, *P* = 0.036).

**Table 2:** Stratification analysis of *XPG* rs873601G>A and risk genotypes with stomach cancer susceptibility

Variables	rs873601 (cases/controls)		Adjusted OR^a^ (95% CI)	*P* a	Risk genotypes (cases/controls)		Adjusted OR^a^ (95% CI)	*P* a
	GG/GA	AA			0	1–2		
Median age, year								
≤59	249/275	84/85	1.05 (0.72–1.53)	0.812	64/92	269/268	1.44 (0.97–2.14)	0.070
>59	256/326	103/85	**1.54 (1.05–2.25)**	**0.028**	81/108	278/303	1.13 (0.77–1.65)	0.538
Gender								
Females	146/170	54/55	1.06 (0.65–1.75)	0.810	45/55	155/170	1.04 (0.62–1.74)	0.891
Males	359/431	133/115	1.37 (1.00–1.89)	0.052	100/145	392/401	**1.40 (1.01–1.93)**	**0.043**
Smoking status								
Never	310/275	117/86	1.14 (0.80–1.64)	0.463	98/82	329/279	0.94 (0.65–1.36)	0.737
Ever	195/326	70/84	1.43 (0.95–2.15)	0.085	47/118	218/292	**1.83 (1.20–2.80)**	**0.005**
Pack‐year								
0	310/275	117/86	1.14 (0.80–1.64)	0.463	98/82	329/279	0.94 (0.65–1.36)	0.737
≤27	100/201	33/49	1.29 (0.73–2.29)	0.385	24/75	109/175	**1.83 (1.02–3.33)**	**0.045**
>27	95/125	37/35	1.52 (0.82–2.84)	0.185	23/43	109/117	1.87 (0.97–3.61)	0.062
Drinking status								
Never	394/420	145/119	1.26 (0.92–1.73)	0.144	108/140	431/399	**1.41 (1.02–1.94)**	**0.036**
Ever	111/181	42/51	1.27 (0.75–2.13)	0.377	37/60	116/172	0.97 (0.57–1.64)	0.896
BMI								
<18.5	38/4	15/1	1.76 (0.17–18.58)	0.640	13/1	40/4	0.87 (0.08–9.37)	0.907
18.5–24.0	301/195	122/49	1.62 (1.11–2.38)	0.013	87/61	336/183	1.30 (0.89–1.90)	0.170
>24.0	166/402	50/120	1.00 (0.68–1.47)	0.993	45/138	171/384	1.33 (0.90–1.97)	0.148
Tumour sites								
Cardia	148/601	51/170	1.17 (0.79–1.74)	0.433	43/200	156/571	1.16 (0.78–1.73)	0.472
Non‐cardia	357/601	136/170	**1.36 (1.01–1.82)**	**0.041**	102/200	391/571	1.33 (0.98–1.81)	0.064

a Adjusted for age, gender, BMI, smoking and drinking status.

OR: odds ratio; CI: confidence interval; BMI: body mass index.

The results were in bold, if the 95% CI excluded 1 or P<0.05.

## Discussion

In the current hospital‐based case–control study, we assessed the association between four potentially functional SNPs in the *XPG* gene and the risk of stomach cancer in Chinese Han population. We found a significant association between *XPG* rs873601 G>A polymorphism and stomach cancer susceptibility, especially in older subjects and subjects with non‐cardia stomach cancer. We also observed the individuals carrying one or two risk genotypes had a significantly increased stomach cancer risk, especially among ever smokers and men.

*XPG* gene, also known as *ERCC5*, is located at 13q33 and consists of 15 exons. There are at least 2430 reported SNPs in the gene region (http://www.ncbi.nlm.nih.gov/SNP). XPG is a structure‐specific endonuclease. It participates in both the transcription‐coupled repair 19 and the global genomic NER 20 steps, which are critical for correcting the excision repair deficiency. As reported, XPG may also play important roles in other cellular processes, such as RNA polymerase II transcription and transcription‐coupled DNA repair 21. Xeroderma pigmentosum group G can excise damaged oligonucleotide by cleaving the 3′ damaged site during NER. The XPF/ERCC1 complex may participate in the 5′ incision, and stabilize the binding of DNA repair complex to damaged DNA 22, 23, 24.

Polymorphism in the *XPG* gene may be associated with cancer susceptibility. In a recent study including a total of 878 colorectal cancer (CRC) patients and 884 controls, Du *et al*. 25 found a significant increased CRC risk for the carriers of *XPG* rs17655 CG/GG genotypes (OR = 1.39, 95% CI = 1.14–1.69). In the same study, a meta‐analysis was also performed on the association of the SNP with CRC risk, with a total of 2649 CRC cases and 2848 controls included. The association between *XPG* rs17655 and CRC risk was replicated under the dominant model (CG/GG *versus* CC: OR = 1.35, 95% CI = 1.20–1.51) in the pooled analysis. Thus far, several studies have been carried out to investigate the association between *XPG* polymorphisms and stomach cancer susceptibility. Hussain *et al*.^13^ performed a population‐based study with 196 stomach cancer cases and 397 controls. They found that the rs2227869G>C polymorphism was associated with decreased stomach cancer susceptibility, while no association was found for the rs1047768 T>C and rs17655 C>G. Canbay *et al*. 14 conducted a study involving only 40 stomach cancer cases and 247 controls, and failed to find any association between rs17655 C>G polymorphism and stomach cancer susceptibility. He *et al*. 16 genotyped three potentially functional *XPG* SNPs (rs2094258 C>T, rs2296147 T>C and rs873601G>A) in 1125 stomach cancer cases and 1196 controls. They found carriers of the rs873601 A had a significantly increased stomach cancer risk. Consistently, they also demonstrated that the A allele were significantly associated with reduced mRNA expression level of *XPG* gene. Duan *et al*. 15 focused on SNPs in the promoter region of *XPG* gene and genotyped the rs751402 C>T and rs2296147 T>C polymorphisms in a total of 403 stomach cancer cases and 403 controls. Both of the two polymorphisms were shown to significantly increase stomach cancer risk. In another study with 337 stomach cancer cases and 347 controls, Yang *et al*. 17 found that the rs2296147 T>C polymorphism was associated with decreased stomach cancer risk, while the rs2094258 C>T polymorphism was associated with increased stomach cancer risk. However, no significant association was found for the rs873601G>A polymorphism. In this study with 692 stomach cancer cases and 771 controls, we found that only the *XPG* rs873601G>A polymorphism is associated with slightly increased stomach cancer risk. No association with stomach cancer risk was found for the remaining SNPs, which may be partially ascribed to the weak effect of each SNP. Besides, the sample size in this study was moderate and might not be large enough to detect relatively weak association. Moreover, the positive findings by others with smaller sample size may be due to a chance.

Despite the significant findings, several limitations should be addressed. First, the sample size is moderate, with only 692 cases and 771 controls included. The relative small sample size might not be able to reveal some weak gene‐disease association and gene‐environment interactions. Second, some valuable information on other exposures for individual participants was missing, such as *H. pylori* infection, occupation and local environmental factors, diet, physical activity. Third, only four potentially functional SNPs were included in this study, and SNPs from the coding and the intron regions that may also be related to stomach cancer risk were omitted. Finally, functional analysis was not performed for the studied SNPs.

In conclusion, this study provided evidence of the associations between four potentially functional SNPs in the *XPG* gene and the risk of stomach cancer. In particular, we found that the *XPG* rs873601G>A polymorphism was associated with slightly increased stomach cancer risk in Chinese Han population. In the future, prospective studies with different ethnicities and larger sample size as well as investigations into mechanism are warranted to validate the role of *XPG* SNPs in stomach cancer carcinogenesis and explore the underlying mechanism.

## Conflicts of interest

None.

## Supporting information


**Table S1** Frequency distribution of selected characteristics in stomach cancer cases and controls.
