# Supplementary material for: Association between XPG polymorphisms and stomach cancer susceptibility in a Chinese population
Source: J Cell Mol Med. 2016 Jan 28;20(5):903–8. doi: 10.1111/jcmm.12773 (PMC4831351; doi:10.1111/jcmm.12773)
Supplement: Supplementary file 1 — Table S1 Frequency distribution of selected characteristics in stomach cancer cases and controls. [file JCMM-20-903-s001.doc]

| **Supplemental Table 1.** Frequency distribution of selected characteristics in stomach cancer cases and controls | | | | | |
| --- | --- | --- | --- | --- | --- |
| Variables | Cases (n=692) | | Controls (n=771) | | *P ^a^* |
|  | No | % | No. | % |  |
| Age range, yr | 24-85 | | 23-87 | | 0.864 |
| Mean ± SD | 59.22 ± 11.05 | | 59.71 ± 11.35 | |  |
| ≤ 50 | 134 | 19.36 | 151 | 19.58 |  |
| 51-60 | 225 | 32.51 | 241 | 31.26 |  |
| 61-70 | 226 | 32.66 | 248 | 32.17 |  |
| >70 | 107 | 15.46 | 131 | 16.99 |  |
| Gender |  |  |  |  | 0.906 |
| Males | 492 | 71.10 | 546 | 70.82 |  |
| Females | 200 | 28.90 | 225 | 29.18 |  |
| Smoking status |  |  |  |  | <0.0001 |
| Never | 427 | 61.71 | 361 | 46.82 |  |
| Ever | 265 | 38.29 | 410 | 53.18 |  |
| Pack-years |  |  |  |  | <0.0001 |
| 0 | 427 | 61.71 | 361 | 46.82 |  |
| ≤ 27 (mean) | 133 | 19.22 | 250 | 32.43 |  |
| > 27 (mean) | 132 | 19.08 | 160 | 20.75 |  |
| Drinking status |  |  |  |  | 0.0005 |
| Yes | 153 | 22.11 | 232 | 30.09 |  |
| No | 539 | 77.89 | 539 | 69.91 |  |
| BMI |  |  |  |  | <0.0001 |
| <18.5 | 53 | 7.66 | 5 | 0.65 |  |
| 18.5-24.0 | 423 | 61.13 | 244 | 31.65 |  |
| >24.0 | 216 | 31.21 | 522 | 67.70 |  |
| Tumor sites |  |  |  |  |  |
| Cardia | 199 | 28.76 | / | / |  |
| Non-cardia | 493 | 71.24 | / | / |  |
| BMI, body mass index.  ^a^ Two-sided *χ^2^* test for distributions between stomach cancer cases and controls. | | | | | |
